# Effect of Sex and Body Mass Index on Children’s Physical Activity Intensity during Free Play at an Indoor Soft Play Center: An Exploratory Study

**DOI:** 10.3390/ijerph14091052

**Published:** 2017-09-12

**Authors:** Michelle A. Jones

**Affiliations:** East Park Terrace, Southampton Solent University, Southampton SO14 OYN, UK; michelle.jones@solent.ac.uk; Tel.: +44-23-8201-6831

**Keywords:** play, physical activity, children, play centers, soft play

## Abstract

**Background**: Indoor soft play can provide a safe but exciting physical activity opportunity regardless of environmental conditions. Relatively little is known about the quality or quantity of physical activity engaged in by children during indoor free soft play. The aim of this study was to evaluate the contribution indoor free soft play can make in enabling children to meet physical activity guidelines and to evaluate the effects of sex and body mass index category. **Methods**: Seventy-two boys and girls aged five to 10 years engaged in un-controlled indoor free soft play with a mean duration of 120.7 (27.1) min, during which physical activity was monitored using Actigraph accelerometers. **Results**: Children spent an average of 61.7 (24.2) min engaging in moderate to vigorous physical activity (MVPA) and 51.4% (*n* = 37) achieved the recommended 60 min of MVPA through the single visit to the indoor soft play center. Boys (68.3 (25.7) min) engaged in significantly (*p* < 0.05) more MVPA than girls (55.8 (21.4) min). Normal weight (65.7 (23.3) min) children engaged in significantly more MVPA than overweight children (48.0 (18.9) min). **Conclusions**: Attendance at a soft play indoor center has the potential to support children to engage in sufficient MVPA and overcome environmental factors that can restrict physical activity opportunities.

## 1. Introduction

Sufficient quantity and quality of physical activity has important physical and psychological health outcomes (e.g., adiposity, musculoskeletal health, cardiovascular health, metabolic profile and mental health) during childhood and likely into adult years [1]. International physical activity guidelines identify the need for children to engage in daily moderate to vigorous physical activity (MVPA) for at least 60 min [2]. Many young people do not meet the current guidelines of 60 min daily MVPA; the analysis of active healthy kids report cards across 15 global countries identified 10 countries that scored grade D (20–39% meet physical activity guidelines) to grade F (<20% meet physical activity guidelines) [3]. A decline in physical activity trajectories has been observed from children aged seven years rather than from adolescence, as commonly perceived [4]. Children’s physical activity can involve a variety of structured and unstructured activities and their natural tendency involves participation in sporadic, intermittent and fun activities [5]. Unstructured physical activity, or active play, in children’s free time could be a major contributor to total physical activity levels [6,7,8]. Furthermore, active play has the potential to contribute to other child development goals and is recognized as a basic right of every child by the United Nations high commission [9]. Whilst there is not a standard definition of play, its commonly agreed-upon characteristics are freely chosen, personally directed, intrinsically motivated and spontaneous.

Physical activity and play in children is influenced by environmental factors. Children’s activity levels peak in summer and decline in the winter [10,11] and higher outdoor time is related to higher engagement in MVPA [12]. Rich et al. [11] concluded that there was sufficient evidence of the need for interventions to increase physical activity in children during the winter. Likewise, children are more likely to be active on weekends and outside of school time in comparison to during school [13]. Brockman [6] suggested that the after-school period, when children have greater freedom of choice, seems to be a critical period for active play. The United Kingdom National Institute for Health and Care Excellence [14] identifies the need for local indoor and outdoor opportunities for unstructured, spontaneous play.

In recent decades, commercial indoor soft play centers have emerged across western countries as independent centers or as add-ons to restaurants, ferries, service stations and family public houses including in the United Kingdom, Sweden and the United States of America. McKendrick et al. [15,16] described indoor play centers as establishments made safe to attract parents but exciting and risky enough to engage children. Younger children aged two to ten years most frequently engage in indoor soft play [17]. Parents are important gatekeepers [18] and indoor play centers become an essential choice for children’s unstructured free play due to weather, dark nights, limited play space or dangerous neighborhoods [19]. Indoor soft play centers are most frequently used on weekends and after school as a venue for active play.

Brockman et al. [9] identified that a relatively neglected area of research is physical activity obtained through informal play in any environment. Whitehurst et al. [20] monitored the heart rate of five- to ten-year-olds during active free play in an indoor soft play center and identified that the mean heart rate was 77% of the maximum throughout the duration of play. Heart rate as an index of MVPA is limited in the context of an exciting play environment. Current evidence supports the notion that active play during school breaks [21], after school [6] and on weekends can make a major contribution to children meeting physical activity guidelines [22,23]. The quantity of MVPA does seem to be impacted by sex, with boys achieving more MVPA minutes through play than girls [6,23]. Howe et al. [24] identified higher energy expenditure above resting level during games in a free play environment for healthy weight compared to overweight children. The provision of play equipment increases the time children spend in MVPA [21,25,26,27], which supports the notion that indoor soft play centers could support children to meet MVPA guidelines. The aim of this study was to evaluate the contribution indoor free soft play can make in enabling children to meet physical activity guidelines and to evaluate the effects of sex and body mass index (BMI) category.

## 2. Materials and Methods

A cross-sectional observation approach was utilized to record physical activity during natural free play in an indoor soft play center. The indoor soft play center provided a range of equipment including play frame, slides, indoor ball play area and disco area. York St John University ethics committee granted ethical approval for the study (Ethics Code: REF: UC/24/2/12/JW). Boys (*n* = 34) and girls (*n* = 38) aged five to 10 years (6.7 (1.6) years) were recruited for the study, using opportunistic sampling.

On entry to the indoor play center parents were asked if they would be willing to consent to their child being included in the study and those that agreed provided written informed consent, whilst verbal assent from the child was also obtained. The child’s age, sex, height and mass were recorded and an accelerometer was fitted under parental supervision. Participants then completed their normal stay at the indoor soft play center and returned to the research group at the end of their session. Data were collected over a period of six weeks after school hours between the months of January and February during the winter season.

Physical activity was assessed using Actigraph GT3 triaxial accelerometers using a 15 s epoch since longer sampling intervals might have masked short intermittent burst of physical activity that are typical for children [28]. A trained research assistant fitted the accelerometer using a suitably sized elastic band at hip level (right iliac crest). Participants wore the Actigraph for the duration of their stay at the indoor play center. Accelerometer data were uploaded using the Actigraph software (Actilife v3.6.0). Duration of sedentary behavior (0 to 25 counts per 15 s), light physical activity (>25 to ≤573 counts per 15 s) and MVPA (>573 counts per 15 s) were estimated using predetermined cut points for children adjusted to account for different epoch durations [29]. Whilst these cut points are typically reported as counts per minute the original data collected by Evenson et al. [29] were recorded using a 15 s epoch and in children aged five to eight years which is relatively comparable to the current study participant characteristics. Trost et al. [30] suggested these cut points provided excellent classification accuracy for MVPA and sedentary behavior and acceptable accuracy for light physical activity and performed well among children of all ages when compared with indirect calorimetry measures in children between five and 15 years old. Thirty minutes of valid accelerometer data per child was required for inclusion in the analysis.

Body mass index (kg/m^2^) was converted to an age and sex specific standard deviation score [31]. Descriptive statistics for time spent in sedentary, light and MVPA and percent of duration of stay were calculated using SPSS version 20.0 (IBM Corporation, Armonk, NY, USA). Data are summarized as mean (SD). Independent *t*-tests were conducted for MVPA, light physical activity and sedentary time to compare between sex (boys/girls), BMI category (normal weight/overweight) and duration of stay (60 to 120 min/121 to 180 min). Alpha was set at *p* < 0.05.

## 3. Results

### 3.1. Physical Activity during Indoor Free Soft Play

Table 1 summarizes the findings. The mean time spent sedentary was 13.1 (11.7) min, which was 10.7% of the stay duration. Light physical activity was engaged in for 47.4 (18.7) min or 38.8% of the stay duration. Children spent an average of 61.7 (24.2) min engaging in MVPA, which equates to 51.5% of the stay duration. Of the 72 children, 51.4% (*n* = 37) achieved the recommended 60 min of MVPA through the single visit to the indoor soft play center. There was no significant relationship between age and the duration of stay, sedentary time, light physical activity or time spent in MPVA (*p* > 0.05).

### 3.2. Effect of Duration of Stay on Physical Activity during Indoor Free Soft Play

Table 1 summarizes the findings. Time at the indoor soft play center varied from 47 to 180 min amongst participants. Two children spent <60 min in the indoor soft play center, 30 children spent between 60 to 120 min and the remaining 40 children spent between 121 to 180 min at the indoor soft play center. There was a significant relationship between time spent at the indoor play center and time spent engaging in MVPA (*r* = 0.51, *p* < 0.05). Table 1 indicates that regardless of the duration of the stay, the proportion of time spent in MVPA was similar. Of the 30 children with a duration of 60 to 120 min, 34.4% (*n* = 11) met the recommended 60 min of MVPA through the single visit to the indoor soft play center, whereas of the 40 children with a duration of 121 to 180 min, 68.4% (*n* = 26) met the guideline.

### 3.3. Effect of Sex on Physical Activity during Indoor Free Soft Play

Table 1 summarizes the findings. There was no significant difference in sedentary time between boys and girls (*p* > 0.05). Girls spent a significantly greater amount of time engaging in light physical activity in comparison to boys (*p* < 0.05). Boys engaged in significantly more MVPA than girls (*p* < 0.05). The duration of stay was similar between boys and girls, and boys spent a higher proportion of time at the indoor soft play center engaging in MVPA than girls. Of the 34 boys, 64.7% (*n* = 22) achieved the recommended 60 min of MVPA through the single visit to the indoor soft play center, whereas 39.5% (*n* = 15) of the 38 girls did.

### 3.4. Effect of BMI on Physical Activity during Indoor Free Soft Play

Table 1 summarizes the findings. International BMI cut-off points were applied and 48 children were normal weight, 19 were overweight and 5 were obese. A significant difference between normal and overweight children was identified in MVPA (*p* < 0.05) but not in sedentary time (*p* > 0.05) or light physical activity (*p* > 0.05). Of the 48 normal weight children, 62.5% (*n* = 30) achieved the recommended 60 min of MVPA through the single visit to the indoor soft play center, whereas 21.1% (*n* = 4) of the 19 overweight children did. Reviewing the data in Table 1, overweight children had both a lower duration of stay at the indoor play center and spent a smaller proportion of time at the play center engaging in MVPA.

## 4. Discussion

The aim of this study was to evaluate the contribution indoor free soft play can make in enabling children to meet physical activity guidelines and to evaluate the effects of sex and BMI category. The mean time spent engaging in MVPA was 61.7 min and 51.4% of children achieved the full recommended 60 min of daily MVPA during their visit to the soft play indoor center. There is little directly related data on active play in soft play indoor centers, but the current findings support the high intensity through heart rate measurement during play observed by Whitehurst et al. [20]. In simulated free play, Howe et al. [24] identified most games generated MVPA and this supports the notion of active play contributing to MVPA guidelines in children. Escalante et al. [32] proposed that larger play areas resulted in greater MVPA than smaller play areas, and since soft play indoor centers tend to be large this also supports them as a venue for MVPA. In an observational study, Farley et al. [33] reported that when children were given a choice during school recess play, they congregated in areas with fixed play equipment and engaged in physically active play. The finding provides support that active play, despite its sporadic and intermittent nature, is an important option to support young children in meeting physical activity guidelines [6,7].

The current study observed children’s free play in a natural environment without intervention and the mean proportion of time spent engaging in MVPA was 51.5%, although boys (58.1%) engaged in MVPA for a higher proportion of time than girls (45.7%). The proportion of time spent in MVPA at the soft play indoor center is within a similar range to that reported during school recess time, which has been reported in the region of 35% to 41% [34], 46% (lunch break) to 59% (recess) [35] and 51% (lunch) to 56% (recess) [26]. Previous data seems to indicate the longer the break, the lower the proportion of time spent engaging in MVPA, for instance when comparing longer lunch breaks to shorter recess periods. The mean duration of stay at the soft play indoor center was 122 min, which is higher than a typical lunch or recess break, and despite this children maintained a high proportion of time engaged in MVPA. Furthermore, analysis revealed that whilst the absolute quantity of MVPA was related to the duration of stay at the indoor soft play center, the proportion of time in MVPA was similar between a stay of 60–120 min (50.5%) compared to 121 to 180 min (52.0%). During school play times, where game equipment is provided, the time spent in MVPA is increased [26,27,36] and this further supports the notion that access to high quality play equipment can help support children in engaging in active play which contributes to achieving physical activity guidelines. In a systematic review of 53 studies, Ridgers et al. [21] identified that overall facility provision was associated with physical activity during school recess free play and suggested that increasing access to different facilities during recess breaks may benefit children’s engagement in physical activity.

In line with other literature related to physical activity during play [23,26,37,38], boys engaged in significantly more MVPA and spent a greater proportion of their time in MVPA. Ridgers et al. [21], in a systematic review, also found boys were more physically active than girls during recess play and proposed boys may view the recess as an opportunity to play competitive games whereas girls may view it as a time to socialize with friends. Since the current age range of children was five to ten years, this finding reinforces the concept that children engage in natural free play differently depending upon sex, prior to biological factors intervening. Despite girls participating in less MVPA, the mean duration of MVPA in girls was 55.8 min and 39.5% of the girls did meet the recommended 60 min MVPA in the single visit. 

Normal weight children engaged in significantly more MVPA and spent a greater proportion of their time in MVPA than overweight children. Nonetheless, overweight children engaged in 48.0 (18.9) min of MVPA. There is little consistent evidence regarding the impact of overweight on MVPA during free play; Howe et al. [24] also identified significantly lower energy expenditure in overweight children during simulated free play and Stratton et al. [39] indicated overweight boys, but not girls, were significantly less active during primary school recess. The potential impact of a single cut-off being utilized to classify MVPA needs considering when interpreting this finding since it has been suggested that the threshold for MVPA may be lower in overweight individuals; for instance, relative intensity classification may be more appropriate since the same absolute activity count may require greater effort due to the higher body mass and/or lower relative fitness [40]. These findings indicate the need for care when considering physical activity via active free play as part of weight intervention programs in children, although physical activity as a core component of interventions has been suggested as an effective approach at reducing BMI in primary-aged children [41]. Furthermore, the International Study of Childhood Obesity, Lifestyle and the Environment (ISCOLE), which analyzed data from 6539 children in a cross-sectional sample across 12 global study sites, suggested greater MVPA is associated with a lower odds of obesity independent of sedentary behavior; it was suggested attaining at least 55 min per day was associated with lower obesity in this multinational sample of children [42]. This could suggest that active play may be a positive addition to programs aiming to support young people in being active as part of weight control programs.

The most important limitation of the current study is the lack of 24 h measurement of physical activity. This could mean that after attendance at the soft play indoor center, a compensatory decline in physical activity was possible; for instance, Veitch et al. [8] identified no difference in MVPA between children within the highest and lowest tertiles for frequency of playing when measuring physical activity across a whole day, and Ridgers et al. [43] reinforced the concept on a compensatory hypothesis whereby children compensate for physical activity and sedentary time between days. Recent research has suggested a similar classification of sedentary time and MVPA when estimated with hip- versus wrist-worn accelerometers across daily physical activity [44]; nonetheless, the nature of the equipment at the soft play center could have led to some upper body activities (e.g., swing balls in the play frame) not being monitored. A further limitation is that the novel factor of the soft play indoor center could have contributed to the higher proportion of time engaged in MVPA; it is unclear if regular attendance (e.g., a soft play after school club) at a soft play indoor center would sustain such high levels of MVPA. Finally, the opportunistic sample method provides no insight into the type of children who attend soft play indoor centers and the sample could have been biased towards active children and motor skill proficiency was not considered.

## 5. Conclusions

Attendance at a soft play indoor center has the potential to support children in engaging in sufficient MVPA and overcoming environmental factors that can restrict physical activity opportunities. Future research should consider evaluating whether indoor free soft play sustains high levels of MVPA over a prolonged period, e.g., via after-school or weekend clubs. Furthermore, a greater appreciation of the type and nature of physically active play and how they are influenced within social contexts in indoor soft play environments would be beneficial, for instance through the application of a tool such as the system for observing children’s activity and relationships during play tool (SOCARP) [45].

## Figures and Tables

**Table 1:** Physical activity intensity and stay duration of boys, girls and by BMI classification (mean (SD)).

Characteristic	Duration of Stay (min)	Sedentary Duration (min)	Light Physical Activity Duration (min)	MVPA Duration (min)	Proportion of Time in MVPA (%)
All children (*n* = 72)	120.7 (27.1)	13.1 (11.7)	47.4 (18.7)	61.7 (24.2)	51.5 (16.8)
Boys (*n* = 34)	118.0 (31.0)	11.4 (10.4)	40.4 (17.5) ^a^	68.3 (25.7) ^a^	58.1 (15.9)
Girls (*n* = 38)	123.0 (23.2)	14.6 (12.7)	53.7 (17.8) ^a^	55.8 (21.4) ^a^	45.7 (15.6)
Duration of stay 60 to 120 min (*n* = 30)	101.9 (14.9)	6.6 (5.4)	44.9 (16.0)	50.9 (16.3)	50.5 (15.3)
Duration of stay 121 to 180 min (*n* = 40)	140.1 (16.3)	18.8 (12.9)	51.1 (19.6)	72.4 (25.0)	52.0 (17.9)
Normal Weight (*n* = 48)	123.7 (24.3)	14.2 (12.9)	45.7 (17.1)	65.7 (23.3) ^b^	53.3 (16.1)
Overweight (*n* = 19)	105.8 (28.3)	10.1 (8.3)	48.6 (21.6)	48.0 (18.9) ^b^	47.4 (18.2)

^a^ Significant difference between boys and girls (*p* < 0.05); ^b^ Significant difference between normal weight and overweight (*p* < 0.05).
